# Physical Health Profile and Associated Behavior During the COVID-19 Pandemic in Patients With Bipolar Disorder

**DOI:** 10.3389/fpsyt.2021.759694

**Published:** 2021-12-06

**Authors:** Jon Dyg Sperling, Nina Dalkner, Christina Berndt, Eva Fleischmann, Michaela Ratzenhofer, Julia Martini, Andrea Pfennig, Michael Bauer, Eva Reininghaus, Maj Vinberg

**Affiliations:** ^1^Mental Health Center, Northern Zealand, Copenhagen University Hospital – Mental Health Services CPH, Copenhagen, Denmark; ^2^Department of Clinical Medicine, University of Copenhagen, Copenhagen, Denmark; ^3^Department of Psychiatry and Psychotherapeutic Medicine, Medical University Graz, Graz, Austria; ^4^Department of Psychiatry & Psychotherapy, Faculty of Medicine, Carl Gustav Carus University Hospital, Technische Universität Dresden, Dresden, Germany

**Keywords:** physical health, anxiety, bipolar disorder, COVID-19 pandemic, behavioral changes

## Abstract

**Background:** The COVID-19 pandemic has led to an increased psychological strain on public mental health and may impact behavioral, mental, and physical health, presumably with effects on patients with severe mental disorders. This study examines pandemic-related physical and mental health and (compensatory) behavioral changes, in patients with BD as compared to healthy control individuals.

**Method:** Physical and mental health and self-reported changes in daily structure and behavior due to the pandemic were assessed using a self-constructed questionnaire and the brief symptom inventory (BSI) in Germany, Austria, and Denmark in individuals with BD and a healthy control group.

**Results:** The present study included 118 individuals with BD and 215 healthy controls. Individuals with BD reported statistically significant higher physical risk burden, increased weight gain, more physical comorbidities, and a decrease in physical activity and they further reported higher rates of COVID-19 testing, had more worries concerning health, and experienced more anxiety but less social distancing.

**Conclusion:** The COVID-19 pandemic seems to have a greater impact on physical health in individuals with BD than in healthy controls. Individuals with BD appear to be having more difficulties compensating their behavior due to the pandemic which could amplify the effect of risk factors associated with poorer physical health. This highlights the necessity for optimizing and targeting the overall treatment of both mental and physical health in patients with BD during periods with far-reaching changes such as the COVID-19 pandemic.

**Limitations:** Sampling issues and self-report forms, selectivity (missing elderly, and those lacking access or knowledge of technology).

## Background

The severe acute respiratory syndrome coronavirus 2 (SARS-CoV-2) has spread through the world and is classified by the World Health Organization (WHO) as a pandemic. Scientific evidence from previous pandemics points to an increased risk for negative psychological effects due to isolation through government-initiated lockdowns and social distancing (1). During coronavirus disease 2019 (COVID-19) pandemic, several lockdowns, deconfinement, fear of resurgence, and the balance between isolation vs. return to normality have made daily routines hard to uphold. Loss of daily structure and reduced social contacts were associated with boredom, frustration, and reduced psychological well-being and distress in the general population (2).

A growing literature reports the psychological implications of the COVID-19 pandemic (3–6) and international networks like COVID-MINDS Network (https://www.covidminds.org/) bring together and harmonize European cohorts. A longitudinal study of 2,00,000 Western and Northern Europeans showed that COVID-19-related worries were persistently high until reopening phases and that younger individuals and individuals with psychiatric illness expressed the highest levels of loneliness (7). The latter study concluded that the COVID-19 pandemic has major impact on mental health and recommended that the above-mentioned subgroups should be closely monitored and provided with tailored interventions to avoid long-term negative effects on mental health (7).

Bipolar disorder (BD) ranks as the 17th leading source of disability of all diseases worldwide, with a lifetime prevalence of ~1–2% (8, 9). BD is characterized by recurrent episodes of mania and depression and the age of onset is often in young adulthood (10, 11). Overall, BD is associated with increased rates in all physical disease groups as defined in international classification of diseases, 10th version, except for cancer (12). Further, overweight and obese patients with BD seem to have poorer treatment response, more affective episodes, more depressive symptoms, higher rates of cognitive impairment, prolonged acute treatment, and increased risk of suicide as compared to BD patients with a lower body mass index (BMI) (13–15). The associated reduced expected life span is 8–12 years, with the leading cause of death being from cardiovascular events/diseases (16). Finally, individuals with BD tend to have more sedentary behavior than healthy controls (HC) and given that inactivity is an independent predictor of cardiovascular disease, it has been advised to address this aiming to improve overall physical health (17, 18).

Anxiety symptoms are common in BD as well, often persist in the remitted phases of BD, and seem to act as a trigger for new affective episodes (19). Overall, patients with BD are thus considered as an at-risk population, especially vulnerable to recurrent affective episodes during the pandemic (1). For patients with BD, it has been suggested that targeted interventions and monitoring/interventions for physical health are prudent (20–22). Accordingly, recent recommendations for handling patients with BD during the present pandemic have been developed including validating emotional reactions (e.g., fear, anxiety, and sadness), and monitoring physical health and promote healthy lifestyle choices and health problems arising from confinement (1). However, studies on patients with BD in relation to the COVID-19 pandemic are scarce (23, 24). Thus, more data are needed to develop data- and evidence-driven strategies to reduce the adverse impacts on psychological and physical health during the pandemic and related profound changes of daily life in individuals with BD.

### Aims of the Study

This multicentric study aimed to assess the psychological and physical impact of the COVID-19 pandemic in individuals with affective disorders. The present analysis specifically examined risk factors associated with physical health during the COVID-19 pandemic, and the association with worries/anxiety in individuals with BD. We hypothesize that individuals with BD (vs. HC individuals) show (i) a significantly higher proportion of risk factors associated with poorer physical health, (ii) pandemic-related behavioral and mental changes, and (iii) lesser extent of compensatory behavioral changes in relation to physical activity.

## Methods

### Participants and Procedure

The study was conducted as an online questionnaire survey using the software Lime Survey (https://www.limesurvey.org) in three outpatient clinics from May 2020 until September 2020 (Graz, Austria: 5.5.21–4.6.21; Dresden, Germany: 15.6.20–22.9.20, and Copenhagen, Denmark: 20.6.20–22.9.20. All data was collected pseudo-anonymously using an individual participant's code.

Eligibility criteria for participation were: 18 years or older, a diagnosed psychiatric disorder for patients and the absence of a psychiatric diagnosis for HC or family history and being able to understand the information describing the research study, informed consent, as well as Internet and email access.

This study was conducted in accordance with the Declaration of Helsinki and was approved by the local Ethics Committees (DK H-20209804).

### Questionnaires

Sociodemographic characteristics, physical health status, medical information, and subjective experiences of changes in relation to the COVID-19 pandemic were assessed using a self-constructed questionnaire. The questionnaire was developed by the Department of Psychiatry and Psychotherapeutic Medicine at the Medical University of Graz (67 items). The process of development of the questionnaire was as follows: The information required from the questionnaire was decided upon between the clinical researcher staff made up of psychologists and psychiatrists. Then target respondents were identified and more precise question content and wording formulated. Finally, the questions were put into meaningful order and formatted according to categories and themes.

Physical health risk factors were examined using the following items: Weight and height (for BMI calculation).

“*Do you have any of the following diseases: cardiovascular, diabetes, chronic obstructive pulmonal disease, hepatitis B, hypertension, chronic kidney failure, cancer, rheumatoid illness and other*.” The number was pooled to give a total amount of physical diseases.

“*Have your smoking habits changed (compared to the time before the COVID outbreak)”* (“yes, I smoke less now,” “yes, I smoke more now” or “no, I smoke as much as before the COVID outbreak”).

“*Has your alcohol consumption changed (compared to the time before the COVID outbreak)*” (“yes, less alcohol” vs. “yes, more alcohol” or “no change”).

“*Has your weight changed since the government announced the lockdown (since 23rd March)*” (“yes, I have gained more than 5 kg,” “yes, I have lost more than 5 kg” or “no, no change in weight”).

“*With regards to the aforementioned physical activities, has your level of physical activity now changed compared to before the Coronavirus pandemic?*” (“yes, I was more physically active,” “yes, I was less physically active” or “no, I am as active now as before the Coronavirus pandemic”).

Behavioral indicators were assessed using the following items:

“*To which extent do the following statements apply to you in the last week: I worry about my health?*” (0 = strongly disagree; 1 = slightly disagree; 2 = neither agree nor disagree; 3 = slightly agree; 4 = strongly agree).

“*Have you been tested for COVID-19 at any time since the pandemic began?*” (1 = yes; 2 = no).

“*Has the frequency of your social contacts changed in the last week compared to the time before the coronavirus outbreak*” (0 = “yes, significantly more social contacts”; 1 = “yes, significantly fewer social contacts,”; 2 = “no change”).

“*Has your daily routine and structure changed since the COVID outbreak” (0* = “*yes, more structure*”; 1 = “yes, less structure”; 2 = “no change”). We looked at the group who reported less structure.

Finally, the Brief Symptom Inventory-18 (BSI-18; 18 items) (25) was used to assess psychological symptoms in the anxiety subscale, during the last seven days. The questionnaire is a short version of the Symptom-Checklist-90 Revised. The BSI-18 has a Cronbach alpha value of α = 0.84, which indicates good reliability (26).

### Statistical Analysis

The Statistical Package for Social Sciences was used as a database and to undertake statistical analyses (SPSS, version 26 for windows). To compare patients with BD and HC, comparisons of means with chi-square tests and Mann-Whitney-U tests were conducted. For dichotomous outcomes, multiple logistic regression analyses were performed. For logistic regression analysis, BMI was divided into two groups below (normal weight) and above (overweight) 25 kg/m^2^. The above-described physical risk factors and behavioral indicators were dichotomized and analyzed in using a basic adjusted model 1, including sex and age and additionally education and a fully adjusted model 2 including cohabitation and education. All models were tested for normality, linearity, and homogeneity of variance using a P-Plots, residual plots, and histograms. The level of statistical significance was set at 5% (two-tailed).

## Results

The questionnaires were (partially or fully) completed by 558 participants. Ninety-eight data sets were excluded due to incomplete data and a further 19 were excluded due to only completing demographic data and an additional four due to unclear information given. Finally, 63 had not completed whether they belonged to the patient or the control group. In total, 374 questionnaires were included in the final dataset. In the present study, overall patients with BD (*n* = 118) and control persons (*n* = 215) were included (in total 333).

Specific test values and degrees of freedom are presented only in the table for the purpose of simplification.

### Sociodemographic Characteristics, Physical Health Status, Behavioral Changes, and Anxiety

As seen in Table 1, the study comprised of 118 (35.4%) patients with BD, with a mean age of 43.5 (SD ± 13.2) years, and 215 (64.5%) HC, mean age of 39.8 (SD ± 13.6) years. Patients with BD were significantly older (*p* = 0.01), had a lower education level (*p* < 0.001), and lived alone more often (*p* < 0.004). There were no statistically significant differences in sex distribution. Age of onset was 34.0 years (SD ± 11.0) and illness duration 9.6 years (SD ± 9.5). Finally, 39% of the patients with BD registered an active lithium treatment. Concerning any other medical treatment, considerably more patients (95%) than HC (22%) reported a regular intake.

**Table 1:** Sociodemographic characteristics, physical health status, COVID-19 behavioral changes, and anxiety scores in patients with bipolar disorder (BD) and healthy control individuals (HC), mean (M) and in brackets standard deviation (SD).

	**BD (*n* = 118)**	**HC (*n* = 215)**	***P***	**test**
Age, years (M, SD)	43.5 (13.2)	39.8 (13.6)	0.01	Mann-Whitney
**Sex, (n, %)**
Female	80 (68%)	148 (69%)	0.845	Chi^2^ = 0.038, df = 1
Male	38 (32%)	67 (31%)		
**Study center (n, %)**
Copenhagen	56 (47%)	2 (1%)		
Dresden	26 (22%)	40 (19%)		
Graz	36 (31%)	173 (80%)		
Education (n, %)			<0.001	Chi^2^ = 51.019, df = 5
GCSE/O levels	5 (4%)	10 (5%)		
Apprenticeship	26 (22%)	21 (10%)		
A-Levels/NVQ/HND	34 (29%)	30 (14%)		
Bachelor's Degree	26 (22%)	21 (10%)		
Master's Degree	24 (20%)	97 (45%)		
Doctoral level	3 (3%)	36 (16%)		
Relationship (n, %)			0.004	Chi^2^ = 15.235, df = 3
Single	35 (30%)	48 (22%)		
In a relationship	33 (28%)	75 (35%)		
Married	37 (31%)	87 (40%)		
Divorced/widowed	13 (11%)	5 (3%)		
Active lithium treatment (n, %)	46 (39%)	0 (0%)		
Any regular medical treatment (n, %)	112 (95%)	47 (22%)		
Age of onset (years)	34.0 (SD ± 11.0)	-		
Illness duration	9.6 (SD ± 9.5)	-		
**Physical illness**
BMI	27.6 (7.0)	24.2 (3.5)	<0.001	Mann-Whitney
Somatic diseases			<0.001	Chi^2^ = 29.242, df =3
0	63 (53%)	171 (80%)		
1	45 (38%)	40 (18%)		
2	9 (7%)	5 (2%)		
≥3	3 (2%)	0 (0%)		
**Behavioral changes**
Increase in alcohol intake	9 (10.7%)	34 (18.0%)	0.312	Chi^2^ = 2.331, df. = 2
Increase in smoking habits	12 (25.0%)	10 (33.3%)	0.368	Chi^2^ = 2.001, df. = 2
Weight gain of > 5 kg	26 (22.0%)	11 (5.1%)	<0.001	Chi^2^ = 25.497, df = 2
Change of physical activity		0.007	Chi^2^ = 9.954, df =2
More activity	16 (13.6%)	55 (25.6%)		
Less activity	40 (33.9%)	81 (37.7%)		
**Behavior indicators**
Strongly agrees to worries about health	17 (14.0%)	5 (2.3%)	<0.001	Chi^2^ = 29.284, df = 4
COVID-19-tested	27 (23.0%)	16 (7.0%)	<0.001	Chi^2^ = 16.15, df = 1
COVID-19 related decline in social contacts	42 (35.6%)	131 (60.9%)	<0.001	Chi^2^ = 19.594, df = 2
COVID-19 related less daily structure	40 (33.9%)	65 (30.2%)	0.558	Chi^2^ = 1.168, df = 2
BSI-18, Anxiety	5.6 (4.0)	2.2 (3.0)	<0.001	Mann-Whitney

*BMI, body mass index; N, number; GSCE, general certificate of secondary education; NVQ, national vocational qualification; HND, higher national diploma*.

Looking at physical health, patients with BD had a significantly higher BMI (*p* < 0.001), reported more physical disease *(p* < 0.001), weight gain (*p* < 0.001), and decreased level of physical activity (*p* = 0.007) in comparison to HC. The reported increase in alcohol (*p* = 0.128) and smoking habits (*p* = 0.426) did not differ significantly between the two groups during the pandemic.

Overall, 14% of the patients with BD reported significantly more worries about health vs. 2.3% in the HC group (*p* < 0.001). Statistically significant more patients with BD (23%) had been COVID-19 PCR tested vs. the HC group (7%) (*p* < 0.001). Patients with BD reported a significantly lesser decline in social contacts 35.6 vs. 60.9% as compared to the HC group (*p* < 0.001). There was a significant difference between groups with regard to the BSI-18 anxiety scale, where patients with BD reported higher overall anxiety than HC (5.6 vs. 2.2; *p* < 0.001). Finally, no significant differences between the two groups were seen in relation to having less daily structure during the COVID-19 pandemic (*p* = 0.56).

Physical health status, behavioral changes, and anxiety in patients with BD and HC adjusted for age and sex, basic model 1 and the fully adjusted model 2, additionally adjusted for relationship status and educational level (Table 2).

**Table 2:** Physical health status, behavioral indicators, and anxiety in patients with BD (*n* = 118) and healthy control individuals (*n* = 215) adjusted for age and sex, basic model 1 and the fully adjusted model 2, additionally including relationship status and educational level.

	**Model l**				**Model 2**			
	***P***	**OR**	**95% CI**		***P***	**OR**	**95% CI**	
			**Lower**	**Upper**			**Lower**	**Upper**
**BMI**
BMI ≤ 25		1				1		
BMI > 25	<0.001	2.37	1.48	3.78	<0.001	2.44	1.44	4.15
**Physical diseases**								
0		1				1		
1–9	<0.001	3.34	1.98	5.62	<0.001	2.80	1.53	5.12
**COVID-19 related weight gain of** **>** **5 kg**								
No weight gain or weight loss		1				1		
Weight gain > 5 kg	<0.001	5.56	2.61	11.88	<0.001	6.24	2.60	1.01
**COVID-19 related change of physical activity**								
No change or less activity		1				1		
Increase in physical activity	0.017	0.47	0.26	0.87	0.032	0.47	0.24	0.94
**Agreement with worries about personal health**								
Slight or strong disagreement or no change		1				1		
Slight or strong agreement with personal health worry	<0.001	2.89	1.76	4.74	<0.001	2.95	1.68	5.16
**COVID-19-tested**								
Not tested		1				1		
Have been tested	<0.001	4.14	2.09	8.21	<0.001	5.81	2.54	13.31
**COVID-19 related decline in social contacts**								
No change or more social contacts		1				1		
Decline in social contacts	<0.001	0.36	0.22	0.57	0.002	0.43	0.26	0.73
BSI-18, Anxiety		B				B		
	<0.001	3.57	2..65	4.48	<0.001	3.42	2.46	4.38

*OR, odds ratio; CI, confidence interval; BSI-18, brief symptom inventory-18 items*.

As seen from Table 2, using multiple logistic regression models, patients with BD had a higher odds ratio (OR) (2.44, 95% CI 1.44–4.15) of having a BMI > 25 in the fully adjusted model 2. Patients with BD also had a statistically significant higher OR (OR = 2.8, 95% CI 1.53–5.12) of having a comorbid physical disease and for reported weight gain above 5 kg (OR = 6.24, 95% CI 2.60–1.01). Patients with BD had decreased (OR = 0.47, 95% CI 0.24–0.94) regarding increased exercise activity, they reported increased worries concerning personal health (OR = 2.95, 95% CI 1.68–5.16) and they had conducted more COVID-19 tests (OR = 5.81, 95% CI 2.54–13.31). Fewer patients reported a decline in social contacts (OR = 0.43, 95% CI 0.26–0.73) in comparison to HCs. Finally, the linear regression analysis of the anxiety index showed a coefficient *B* = 3.42 (95% CI 2.46–4.38, *p* < 0.001) using model 2.

## Discussion

The present online survey study comparing 118 patients with BD and 215 HC, reports on mental and physical health status and behavior changes due to the COVID-19 pandemic in three European countries. In accordance with our three hypotheses, the study revealed that patients with BD (i) exhibited statistically significantly poorer overall physical health status and (ii) experienced a higher amount of COVID-19-related behavioral and mental health changes, and (iii) a lesser extent of physical health compensatory behavior such as a higher rate of COVID-19 testing and having more worries than HC.

Overall, these results emphasize the importance of addressing the increased physical health risk profile in patients with BD. This is underlined by the results from a recent Danish register study showing that patients with BD, who had a positive COVID-19 test, had a more unfavorable physical illness outcome in comparison to patients with COVID-19 not having a severe mental illness (27). The present findings support the described recommendations made by Hernández-Gómez et al. (1) and underline that patients with BD should be considered as an at-risk population, presumably, especially vulnerable to relapse during the pandemic. To verify this conclusion finally, studies providing longitudinal data are needed. Nevertheless, the present results call for an increased clinical awareness among the health professionals and an increasing awareness of the importance of health education that should be addressed (28).

This study showed that patients with BD had a statistically significantly more somatic comorbidities and higher BMI which they presumably also had before the pandemic, however, they more often reported >5 kg weight gain during the pandemic and less patients with BD did compensate with an increase in physical activity compared to HC. At the same time, consistently, the results showed that the anxiety levels, worries about health and COVID-19 tests were significantly higher in the patient group. Despite these changes, patients with BD did not report a statistically significant increase in smoking or alcohol habits in comparison with HC. Interestingly, we also found no differences in smoking and alcohol consumption between individuals with BD and controls. The data tendency was that more HCs reported an increase. To our knowledge, no alcohol/smoking recommendation was targeted during the COVID-19 pandemic at our patient category. It could be because patients with BD have a higher baseline consumption of alcohol and cigarettes, or that they were scared for a worse outcome in case of a COVID infection. However, while both groups were similar, they certainly experienced an increase, especially in smoking habits. Surprisingly, a lower percentage of patients with BD reported a decrease in the number of social contacts than controls. Social distancing could be confounded since patients with BD often have fewer social contacts. However, it could also be due to patients with BD not having the needed resources to comply with pandemic guidelines.

These behavioral changes stipulate that patients with BD may be more affected both physically and mentally during the COVID-19 pandemic, and properly exhibit less resources to cope with these challenges. Both, with regard to physical health and in accordance with government guidelines and social distancing.

Clinically, these findings could lead to an increased awareness toward additional efforts to support our patients with BD to maintain daily structure, e.g., start or maintain feasible training routines including awareness on dietary habits to avoid weight increase, motivation to plan social contacts via telephone or Internet on a regular basis, and finally maintain an open door and risk plan creating an easy access to professional help in critical situations.

### Limitations

Results from this multicenter study add knowledge on the physical health profile and pandemic associated behavioral changes in patients with BD from three European countries. However, the following limitations of this online survey must be considered. For the specific assessment of subjective experiences and behavioral changes due to the COVID-19 pandemic, a self-constructed questionnaire was applied to assess subjective experiences. The questionnaire was developed “*ad-hoc*” for this purpose and was not validated. Recall bias cannot be ruled out and due to the self-reported diagnoses, the patient category was in jeopardy of being misplaced and we, therefore, went thoroughly through this variable. Second, manic or depressive symptomatology could affect how the questions were answered and the present psychiatric disease state was not clinically assessed, which could also lead to a bias pertaining to responders and non-responders. Third, we were not able to provide information on and include the number of previous hospitalizations, or whether the patients had a bipolar I or II disorder. Fourth, the cross-sectional design has a limiting factor in that data is only analyzed from one time point and so should not be interpreted as showing causality. Fifth, ~39% of the patients with BD registered an active lithium treatment. The ratio of lithium-treated BD patients in our sample are in line with other samples of patients with BD (15, 29). The age of onset was 34.0 years (SD ± 11.0) which also is in line with other samples (15, 30). Finally, selection bias is expected to lead to missing elderly and those lacking access or knowledge of technology.

## Conclusion

Patients with BD revealed an overall increased burden during the COVID-19 pandemic on their physical health and changes in behavior indicating an increased need for COVID-19 testing, more worries concerning health, a higher amount of anxiety, and less social distancing. Overall, the COVID-19 pandemic seems to have a more severe impact on the physical health of patients with BD and the patients experience more difficulties in coping with the restrictions and behavioral changes that the pandemic has required. Clinically, it is therefore recommended to increase the attention on the overall health status of patients with BD including handling of physical health during the COVID-19 pandemic.

## Data Availability Statement

The raw data supporting the conclusions of this article will be made available by the authors, without undue reservation.

## Ethics Statement

The studies involving human participants were reviewed and approved by DK H - 20209804. Written informed consent for participation was not required for this study in accordance with the national legislation and the institutional requirements.

## Author Contributions

JS, ND, CB, EF, MR, JM, AP, MB, ER, and MV have all contributed in analyzing data, revising manuscript and have approved final submission document.

## Conflict of Interest

MV has within the last three years been a consultant for Lundbeck, Sunovion and Janssen. The remaining authors declare that the research was conducted in the absence of any commercial or financial relationships that could be construed as a potential conflict of interest.

## Publisher's Note

All claims expressed in this article are solely those of the authors and do not necessarily represent those of their affiliated organizations, or those of the publisher, the editors and the reviewers. Any product that may be evaluated in this article, or claim that may be made by its manufacturer, is not guaranteed or endorsed by the publisher.

## References

[1] Hernández-Gómez A Andrade-González N Lahera G Vieta E. Recommendations for the care of patients with bipolar disorder during the COVID-19 pandemic. J Affect Disorders. (2020) 279:117–21. 10.1016/j.jad.2020.09.105 33045553 PMC7834434

[2] Brooks SK Webster RK Smith LE Woodland L Wessely S Greenberg N. The psychological impact of quarantine and how to reduce it: rapid review of the evidence. Lancet. (2020) 395:912–20. 10.1016/S0140-6736(20)30460-8 32112714 PMC7158942

[3] Chacko M Job A Caston F George P Yacoub A Cáceda R. COVID-19-induced psychosis and suicidal behavior: case report. Sn Compr Clin Med. (2020) 2:2391–5. 10.1007/s42399-020-00530-7 33015547 PMC7519695

[4] Ferrando SJ Klepacz L Lynch S Tavakkoli M Dornbush R Baharani R. COVID-19 Psychosis: a potential new neuropsychiatric condition triggered by novel coronavirus infection and the inflammatory response? Psychosomatics. (2020) 61:551–5. 10.1016/j.psym.2020.05.012 32593479 PMC7236749

[5] Smith CM Komisar JR Mourad A Kincaid BR. COVID-19-associated brief psychotic disorder. BMJ Case Rep. (2020) 13:e236940. 10.1136/bcr-2020-236940 32784244 PMC7418683

[6] Huang L Lei W Xu F Liu H Yu L. Emotional responses and coping strategies in nurses and nursing students during Covid-19 outbreak: a comparative study. PLoS ONE. (2020) 15:e0237303. 10.1371/journal.pone.0237303 32764825 PMC7413410

[7] Varga TV Bu F Dissing AS Elsenburg LK Bustamante JJH Matta J. Loneliness, worries, anxiety, and precautionary behaviours in response to the COVID-19 pandemic: a longitudinal analysis of 200,000 Western and Northern Europeans. Lancet Reg Health Eur. (2021) 2:100020. 10.1016/j.lanepe.2020.100020 33870246 PMC8042675

[8] Merikangas KR Jin R He JP Kessler RC Lee S Sampson NA. Prevalence and correlates of bipolar spectrum disorder in the world mental health survey initiative. Arch Gen Psychiat. (2011) 68:241–51. 10.1001/archgenpsychiatry.2011.12 21383262 PMC3486639

[9] Vigo D Thornicroft G Atun R. Estimating the true global burden of mental illness. Lancet Psychiatry. (2016) 3:171–8. 10.1016/S2215-0366(15)00505-2 26851330

[10] Carvalho AF Firth J Vieta E. Bipolar disorder. New Engl J Med. (2020) 383:58–66. 10.1056/NEJMra1906193 32609982

[11] Kessler RC Berglund P Demler O Jin R Merikangas KR Walters EE. Lifetime prevalence and age-of-onset distributions of DSM-IV disorders in the national comorbidity survey replication. Arch Gen Psychiat. (2005) 62:593–602. 10.1001/archpsyc.62.6.593 15939837

[12] Kessing LV Ziersen SC Andersen PK Vinberg M. A nation-wide population-based longitudinal study mapping physical diseases in patients with bipolar disorder and their siblings. J Affect Disorders. (2021) 282:18–25. 10.1016/j.jad.2020.12.072 33387742

[13] Lackner N Bengesser SA Birner A Painold A Fellendorf FT Platzer M. Abdominal obesity is associated with impaired cognitive function in euthymic bipolar individuals. World J Biol Psychiatry. (2015) 17:1–12. 10.3109/15622975.2015.1046917 26068130

[14] Fagiolini A Kupfer DJ Houck PR Novick DM Frank E. Obesity as a correlate of outcome in patients with bipolar I disorder. Am J Psychiat. (2003) 160:112–7. 10.1176/appi.ajp.160.1.112 12505809

[15] Fagiolini A Frank E Scott JA Turkin S Kupfer DJ. Metabolic syndrome in bipolar disorder: findings from the bipolar disorder center for Pennsylvanians. Bipolar Disord. (2005) 7:424–30. 10.1111/j.1399-5618.2005.00234.x 16176435

[16] Kessing LV Vradi E Andersen PK. Life expectancy in bipolar disorder. Bipolar Disord. (2015) 17:543–8. 10.1111/bdi.12296 25846854

[17] Vancampfort D Firth J Schuch F Rosenbaum S Hert MD Mugisha J. Physical activity and sedentary behavior in people with bipolar disorder: A systematic review and meta-analysis. J Affect Disord. (2016) 201:145–52. 10.1016/j.jad.2016.05.020 27235817

[18] Vancampfort D Firth J Schuch FB Rosenbaum S Mugisha J Hallgren M. Sedentary behavior and physical activity levels in people with schizophrenia, bipolar disorder and major depressive disorder: a global systematic review and meta-analysis. World Psychiatry. (2017) 16:308–15. 10.1002/wps.20458 28941119 PMC5608847

[19] Pavlova B Perlis RH Mantere O Sellgren CM Isometsä E Mitchell PB. Prevalence of current anxiety disorders in people with bipolar disorder during euthymia: a meta-analysis. Psychol Med. (2017) 47:1107–15. 10.1017/S0033291716003135 27995827

[20] Brus MJ Solanto MV Goldberg JF. Adult ADHD vs. bipolar disorder in the DSM-5 era. J Psychiatr Pract. (2014) 20:428–37. 10.1097/01.pra.0000456591.20622.9e 25406047

[21] Firth J Siddiqi N Koyanagi A Siskind D Rosenbaum S Galletly C. The lancet psychiatry commission: a blueprint for protecting physical health in people with mental illness. Lancet Psychiatry. (2019) 6:675–712. 10.1016/S2215-0366(19)30132-4 31324560

[22] Krishnan KRR. Psychiatric and medical comorbidities of bipolar disorder. Psychosom Med. (2005) 67:1–8. 10.1097/01.psy.0000151489.36347.18 15673617

[23] Stefana A Youngstrom EA Chen J Hinshaw S Maxwell V Michalak E. The COVID-19 pandemic is a crisis and opportunity for bipolar disorder. Bipolar Disord. (2020) 22:641–3. 10.1111/bdi.12949 32511859 PMC7300615

[24] Youngstrom EA Hinshaw SP Stefana A Chen J Michael K Meter AV. Working with bipolar disorder during the COVID-19 pandemic: both crisis and opportunity. PsyArXiv. 7, p. 1–9. 10.15347/WJM/2020.004

[25] Derogatis LR Fitzpatrick M. The SCL-90-R, the Brief Symptom Inventory (BSI), and the BSI-18. In: Maruish ME, editor. The Use of Psychological Testing for Treatment Planning and Outcomes Assessment: Instruments for Adults. Mahwah: Lawrence Erlbaum Associates Publishers (2004), p. 1–41.

[26] Spitzer C Hammer S Löwe B Grabe H Barnow S Rose M. Die Kurzform des Brief Symptom Inventory (BSI−18): erste Befunde zu den psychometrischen Kennwerten der deutschen Version. Fortschritte Der Neurologie Psychiatrie. (2011) 79:517–23. 10.1055/s-0031-1281602 21870312

[27] Barcella CA Polcwiartek C Mohr GH Hodges G Søndergaard K Bang CN. Severe mental illness is associated with increased mortality and severe course of COVID-19. Acta Psych Scand. (2021) 144:82–91. 10.1111/acps.13309 33894064 PMC8250986

[28] McIntyre RS Berk M Brietzke E Goldstein BI López-Jaramillo C Kessing LV. Bipolar disorders. Lancet. (2020) 396:1841–56. 10.1016/S0140-6736(20)31544-0 33278937

[29] Coello K Kjærstad HL Stanislaus S Melbye S Faurholt-Jepsen M Miskowiak KW. Thirty-year cardiovascular risk score in patients with newly diagnosed bipolar disorder and their unaffected first-degree relatives. Aust N Z J Psychiatry. (2019) 53:651–62. 10.1177/0004867418815987 30518229

[30] Kemp DE Gao K Chan PK Ganocy SJ Findling RL Calabrese JR. Medical comorbidity in bipolar disorder: relationship between illnesses of the endocrine/metabolic system and treatment outcome. Bipolar Disord. (2010) 12:404–13. 10.1111/j.1399-5618.2010.00823.x 20636638 PMC2913710

